# Defect in lysosomal enzyme trafficking and sorting is associated with irreversibility of pulmonary arterial hypertension

**DOI:** 10.3389/fcvm.2026.1763556

**Published:** 2026-05-21

**Authors:** Genfa Xiao, Ying Meng, Zhiming Du, Zhihua Liu, Haiyang Yu, Haobo Wang, Lijun Nie, Xiaoli Liu, Xuemei Lan, Yanyu Duan, Jianxian Xiong, Ziyou Liu

**Affiliations:** 1 Department of Cardiology, Heart Medical Centre, First Affiliated Hospital of Gannan Medical University, Ganzhou, China; 2 Key Laboratory of Prevention and Treatment of Cardiovascular and Cerebrovascular Diseases, Ministry of Education, First Affiliated Hospital of Gannan Medical University, Ganzhou, China; 3 Ganzhou Cardiovascular Rare Disease Diagnosis and Treatment Technology Innovation Center, First Affiliated Hospital of Gannan Medical University, Ganzhou, China; 4 First Clinical Medical College of Gannan Medical University, Ganzhou, China; 5 Department of Cardiovascular Surgery, First Affiliated Hospital of Gannan Medical University, Ganzhou, China

**Keywords:** congenital heart disease-associated pulmonary arterial hypertension, irreversible pulmonary arterial hypertension, lysosomal dysfunction, lysosomal enzyme trafficking and sorting, weighted gene co-expression network analysis

## Abstract

**Background:**

Patients with congenital heart disease-associated pulmonary arterial hypertension (CHD-PAH) have a reversible stage, during which shunt closure reverses PAH. However, PAH is irreversible beyond a certain time point, and the molecular mechanisms underlying the switch from reversible to irreversible PAH remain poorly understood.

**Methods:**

Three transcriptomic datasets were obtained from the Gene Expression Omnibus database. Weighted gene co-expression network analysis (WGCNA) was performed to identify gene modules significantly associated with PAH irreversibility. Serum samples were collected from 40 patients with CHD-PAH, 20 patients with congenital heart disease (CHD), and 20 healthy controls. Protein levels in serum were quantified using enzyme-linked immunosorbent assay.

**Results:**

WGCNA generated 16 gene modules, and one of the modules showing the highest positive correlation with PAH irreversibility was recognized as the key module. GO and KEGG pathway analyses of key module revealed that PAH irreversibility was associated with lysosome. Pathview analysis indicated dysfunctional lysosome in irreversible PAH, including dysregulation of lysosomal enzymes and impaired lysosomal transport and acidification. Comprehensive characterization of lysosome identified a set of downregulated genes, including Gnptab, M6pr, and Arf1, whose expression patterns shifted significantly during the transition from reversible to irreversible PAH. Functional enrichment analysis linked these genes to lysosomal enzyme trafficking and sorting. The downregulation of M6PR, a receptor involved in lysosomal trafficking and sorting, was validated using independent datasets and clinical samples. Additionally, eight lysosomal hub genes were identified, including Ctsd, Ctsk, Ctsb, Ctsa, Pld3, Tpsab1, Lgals3 and Smpdl3a; among them, serum protein levels of CTSD, CTSB, and LGALS3 were significantly elevated in CHD patients with irreversible PAH compared to those with reversible PAH.

**Conclusion:**

This study identified dysfunctional lysosome in irreversible PAH, and the loss of compensation for lysosomal trafficking and sorting may be associated with a switch from reversible to irreversible PAH, thus providing novel insights into the molecular mechanisms underlying irreversible PAH.

## 1 Introduction

Pulmonary arterial hypertension (PAH) is a cardiopulmonary disease with high morbidity and mortality. Changes in the structure and function of pulmonary vascular cells and inflammatory cell infiltration lead to the remodeling of pulmonary vessels and an increase in pulmonary artery pressure, culminating in right ventricular failure and premature death. The most common clinical type of PAH in China is congenital heart disease- associated PAH (CHD-PAH). CHD-PAH is caused by CHD with a left-to-right shunt. Patients with CHD-PAH have a distinct reversible stage during which shunt closure restores normal pulmonary blood flow and reverses pulmonary vascular remodeling and PAH (1). There is a point beyond which the potential benefit of hemodynamic unloading is lost and the pathological remodeling and increase in pulmonary artery pressure are irreversible (1, 2). However, the molecular mechanisms underlying the transition from the reversible to irreversible stage of PAH are poorly understood.

Transcriptomic and proteomic profiling of irreversible PAH have provided insights into its molecular mechanism, biomarkers and therapeutic targets. Proteomic analysis of explanted lungs from CHD patients with irreversible PAH identified biomarkers and therapeutic targets, including caveolin-1, filamin A, cathepsin D, nestin and transgelin (3–5). Van der Feen, D. E. et al. established a monocrotaline (MCT) + shunt induced- PAH model with a dichotomous reversibility response to hemodynamic unloading, mimicking the human PAH associated with CHD (6). In this novel model, hemodynamic unloading reversed PAH caused by MCT + shunt in the early stage, but not in the late stage. The MCT + shunt- induced PAH model provides a valuable tool for studying the pathogenesis of patients with CHD-PAH. RNA sequencing analysis of this novel model revealed that loss of reversibility was associated with a switch from a proliferative to a senescent vascular phenotype, and vascular cell senescence was causal to the irreversible nature of end-stage PAH (6).

Weighted gene co-expression network analysis (WGCNA) is a widely used data mining method, which aims to discover highly correlated co-expressed gene modules. The association of co-expressed modules with phenotypic traits facilitated the identification of crucial co-expressed modules associated with specific phenotypes. WGCNA has been applied to explore co-expressed modules and key genes in locally systemic sclerosis-related PAH (7) and idiopathic PAH (8). We explored the transcriptome of MCT- induced PAH by using the WGCNA and identified two gene modules correlated with PAH severity (9).

The molecular mechanisms underlying the transition from reversible to irreversible PAH are not well understood. In this study, we used the WGCNA method to further analyze the transcriptional profiles of MCT + shunt- induced PAH, and explored the key modules and genes that lead to irreversible PAH, which aimed to provide new insights into the molecular mechanisms of PAH irreversibility.

## 2 Materials and methods

### 2.1 Data preparation and collection of genes associated with lysosome

The raw RNA sequencing dataset and phenotypic traits were derived from the work of van der Feen, D. E. et al. (6) and downloaded from the Gene Expression Omnibus (GEO) with the accession number GSE149899. The GSE149899 dataset comprises RNA sequencing data from rat lung tissues of 6 irreversible PAH,12 reversible PAH and 5 normal controls. One reversible PAH sample (GSM4516520) was excluded because of unclear labeling, and all other samples were included for WGCNA. Two independent datasets with accession numbers GSE149713 and GSE254617, were used to validate the differentially expressed genes (DEGs). The GSE149713 dataset was derived from our previous study and comprised 17 samples, ranging from the onset to progression of PAH (10, 11). The methods used for rat treatment and RNA sequencing were described previously (10). Briefly, 12 rats were randomly assigned to 4 groups and treated with MCT for 1, 2, 3 and 4 weeks and 5 rats served as the controls. The control and MCT-treated rats were sacrificed at the end of weeks 1, 2, 3 and 4 after MCT treatment, and lung tissues were isolated and used for RNA sequencing. The GSE254617 dataset was derived from Hong, J. et al. (12). The GSE254617 datasets (https://www.ncbi.nlm.nih.gov/geo/query/acc.cgi?acc=GSE254617) comprised two batch samples with transcriptomic data of human lung tissues. The batch 2 samples were selected because of their greater sequencing depth (12). Batch 2 included samples from failure donors (controls), CHD patients with irreversible PAH, patients with idiopathic PAH and patients with heritable PAH, and only samples from 16 failure donors and 8 CHD patients with irreversible PAH (sample_id: PHBI_013, PHBI_014, PHBI_045, PHBI_046, PHBI_165, PHBI_166, PHBI_168 and PHBI_065) were chosen for the present study. The genes associated with lysosome were collected from the database of hLGDB (http://lysosome.unipg.it/), a searchable database of human lysosomal genes and their regulation (13), and from lists of lysosomal genes presented in the Gene Ontology and KEGG databases. The genes associated with lysosome were also collected from searches for review articles regarding the human and murine proteomes of the lysosome.

### 2.2 Construction of weighted gene co-expression network

An expression matrix consisting of 22 samples and 20,944 transcripts was used to construct the co-expression network by using the “WGCNA” R package. To meet the scale-free network, the soft-thresholding power β = 6 (scale-free *R*^2^ > 0.8) was used to construct the adjacency matrix. Thereafter, the adjacency matrix was transformed into a topological overlap matrix. Hierarchical clustering and the dynamic tree cut method were used to divide modules and similar modules were merged on the basis of a height cutoff. In the present study, similar modules were merged based on a height cutoff of 0.75 and the minimal number of genes in each gene module was set to 50.

### 2.3 Identification of key modules associated with PAH irreversibility

The irreversible PAH in a rat model is characterized by a sustained increase in mean pulmonary artery pressure (mPAP), right ventricular systolic pressure (RVSP) and right ventricular hypertrophy index (RVHI) and reduced pulmonary artery blood flow acceleration time (PAAT) after hemodynamic unloading (6). The phenotypic traits used for assessing PAH irreversibility include RVSP, mPAP, RVHI and PAAT. The module eigengene is the first principal component of a given module and is a representative of the gene expression in a given module. The correlations between the module eigengenes and phenotypic traits were calculated by using the Pearson correlation coefficient. The modules highly correlated with mPAP, RVSP, RVHI and PAAT were identified as the key modules linked to PAH irreversibility.

### 2.4 Functional enrichment analysis, jvenn analysis and heatmap creation

For functional enrichment analysis, Gene Ontology (GO) and Kyoto Encyclopedia of Genes and Genomes (KEGG) pathway enrichment analyses were performed by DAVID, a database for annotation, visualization, and integrated discovery (https://davidbioinformatics.nih.gov/home.jsp). An adjusted p (p-adj) value <0.05 was viewed as significant enrichment. The enrichment of highly represented transcription factors was conducted by ChEA3 (https://maayanlab.cloud/chea3/). The overlapping genes were determined by jvenn (https://jvenn.toulouse.inra.fr/app/example.html). Heatmap creation and hierarchical clustering were conducted by Morpheus (https://software.broadinstitute.org/morpheus/).

### 2.5 Visualization of the lysosomal signaling pathway

The genes annotated in the lysosomal signaling pathway were extracted from the KEGG pathway database (https://www.genome.jp/kegg/pathway.html). Pathview (https://pathview.uncc.edu/analysis) is a visual tool that is used for pathway-based data integration and visualization of a large variety of biological data. The altered genes involved in lysosomal signaling were visualized by using a modified Pathview. The details of the statistical analysis are described in the figure legends.

### 2.6 Identification of lysosomal hub genes associated with PAH irreversibility

The module membership (MM) and gene significance (GS) were used to identify the hub genes as previously described (9). The genes with |MM|≥ 0.8 and |GS|≥ 0.5 were viewed as the candidate hub genes of a given module. The candidate genes that overlapped in DEGs of irreversible PAH were considered as the hub genes.

### 2.7 Patient recruitment and blood sample collection

The patients and their blood samples were obtained from an ongoing research project entitled ADAMTS4 Assesses the Irreversibility of Pulmonary Arterial Hypertension Associated with Congenital Heart Disease (Number 20212BAG70028). In this study, individuals with congenital heart disease (CHD), CHD-associated pulmonary arterial hypertension (CHD-PAH), as well as healthy volunteers, were prospectively enrolled between January 1, 2020, and the present date. Pulmonary arterial hypertension was defined as a mean pulmonary arterial pressure (mPAP) exceeding 20 mmHg at rest. Patients with other known etiologies of PAH beyond CHD were excluded. Additional exclusion criteria included acute infection, coexisting coagulopathy, hematologic disorders, and current use of targeted PAH therapy or anticoagulant treatment. Healthy volunteers were recruited from hospital staff and regular blood donors, and all reported no history of cardiac or pulmonary diseases and were not receiving any pharmacological therapies. Peripheral venous blood was collected upon admission or on the day prior to surgery. Serum samples were obtained by centrifugation at 2000 rpm for 10 min at 4°C and stored at −80°C within three hours of collection. A total of 80 participants were selected from the cohort, including 40 patients with CHD-PAH, 20 patients with CHD, and 20 healthy volunteers. Among the 40 patients with CHD-PAH, 20 were diagnosed with irreversible PAH. Irreversible PAH was defined by any one of the following three criteria: (1) postoperative hemodynamic non-recovery, defined as mPAP ≥20 mmHg at rest on right heart catheterization one year after successful transcatheter or surgical defect closure; (2) preoperative Eisenmenger syndrome, confirmed by echocardiography and arterial blood gas analysis before intervention; or (3) intraprocedural contraindication to closure, defined as a pulmonary-to-systemic flow ratio (Qp/Qs) > 1.5 and pulmonary vascular resistance (PVR) > 5 Wood units during hemodynamic assessment. The study protocol was approved by the Ethics Committee of the First Affiliated Hospital of Gannan Medical University, and written informed consent was obtained from all participants (Approval Number: LLSC-2023No.235).

### 2.8 Enzyme-linked immunosorbent assay

Serum protein levels of human and rat cathepsin D (CTSD), human and rat cathepsin B (CTSB), human cathepsin K (CTSK), human galectin-3 (LGALS3), human tryptase (TPSAB1), human mannose-6-phosphate receptor (M6PR), human N-terminal pro-B-type natriuretic peptide (NT-proBNP) and human C-reactive protein (CRP) were quantified using enzyme-linked immunosorbent assay (ELISA) kits (Cloud-Clone Corp., China) according to the manufacturer's instructions. In brief, standards and samples were added to microplate wells precoated with a biotin-conjugated antibody specific to the target antigen. Subsequently, avidin conjugated to horseradish peroxidase (HRP) was added to each well and incubated under controlled conditions. Following incubation, tetramethylbenzidine (TMB) substrate solution was added to initiate the enzyme-substrate reaction. The reaction was terminated by the addition of sulfuric acid solution, and the resulting color change was measured using a microplate reader (Thermo Scientific, USA) at an optical density of 450 nm. The concentration of each protein in the samples was determined by interpolating the sample absorbance values against the standard curve generated from known concentrations.

### 2.9 Cell culture and siRNA transfection

Rat pulmonary artery smooth muscle cells (PASMCs, CP-R342, Procell, Wuhan, Hubei, China) were cultured in DMEM/F-12 medium supplemented with 10% fetal bovine serum (FBS) and 1% penicillin-streptomycin at 37°C with 5% CO₂. For small interfering RNA (siRNA) transfection, cells were seeded in 6-well plates one day prior to transfection to achieve 50%–70% confluence. Small interfering RNA targeting rat M6PR (siM6PR, sense sequence: 5-UUAGCUGUCCUUUCUCUUCCU-3) were chemically synthesized as double-stranded RNA duplexes by Genepharma,Shanghai,China. Transfection was performed using Lipofectamine RNAiMAX (Invitrogen, USA) according to the manufacturer's protocol, with a final siRNA concentration of 50 nM. Cells were harvested 48 h post-transfection for RNA extraction.

### 2.10 RT-qPCR

Total RNA was extracted from PASMCs using TRIzol reagent (15596026CN, Thermo Fisher) and reverse transcribed with the PrimeScript RT reagent Kit (RR037A, Takara). qPCR was performed using TB Green® Premix Ex Taq II (RR820Q, Takara) on a QuantStudio 3 real-time fluorescent qPCR system (Thermo Fisher). Primers: M6PR: forward 5-AGGAAGAGAAAGGACAGCTAA-3, reverse 5-UUAGCUGUCCUUUCUCUUCCU-3; GAPDH: forward 5- ACATCATCCCTGCATCCACT −3, reverse 5- GCGGCATGTCAGATCCACAAC-3. Relative mRNA expression was calculated using the 2^⁻*ΔΔ*Ct^ method.

### 2.11 Edu proliferation assay

Cell proliferation was evaluated using the BeyoClick™ EdU-555 Kit (C0075S, Beyotime, China). The indicated treated PASMCs were seeded into 6-well plates and incubated with 10 μM EdU for 2 h at 37°C, fixed with 4% paraformaldehyde for 15 min, and permeabilized with 0.3% Triton X-100 for 15 min. Click reaction was performed with Click Reaction Solution containing Azide 555 for 30 min at room temperature in the dark. Nuclei were stained with Hoechst 33,342. EdU-positive (red) and Hoechst-positive (blue) cells were visualized under a fluorescence microscope and quantified from random fields per well. The proliferation rate was expressed as the percentage of EdU-positive cells relative to total nuclei.

### 2.12 Transwell assay

Cell migration was assessed using Transwell chambers (8-µm pore size). The treated PASMCs (1 × 104 cells) were resuspended in serum-free medium and loaded into the upper chamber of a Transwell 24-well cell culture chamber (3,378, Corning Inc., Corning, NY, USA). The lower chamber was loaded with 600 μL of complete medium containing serum. After 24 h, migrated cells were fixed, stained with crystal violet, and counted on each membrane.

### 2.13 Senescence-associated β-galactosidase (SA-β-Gal) staining

Cellular senescence was assessed using a SA-β-Gal Staining Kit (G1580, Solarbio Science and Technology Co., Ltd) according to the manufacturer's protocol. Briefly, PASMCs cultured in 6-well plates were washed with PBS, fixed with β-Gal Fixative for 15 min at room temperature, and washed three times with PBS. Staining Working Solution (1 mL/well) was added, and plates were sealed and incubated at 37°C for 12–24 h. SA-β-Gal staining was observed under an optical microscope, and blue-stained positive cells were counted. For quantification, random fields per well were examined, and the percentage of SA-β-Gal-positive cells was calculated.

## 3 Results

### 3.1 Construction of the gene co-expression network

**Figure 1:**
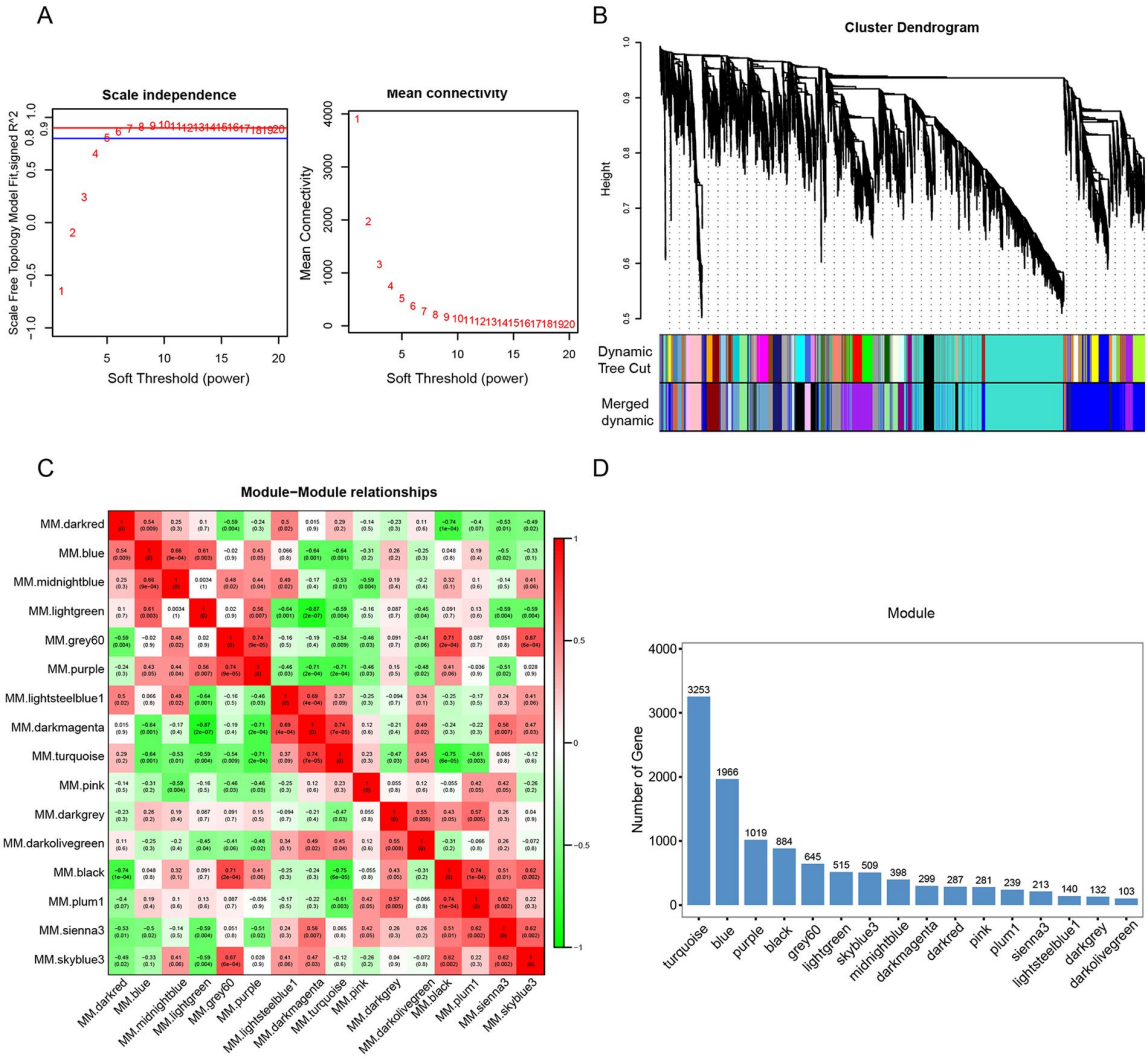
Construction of the gene co-expression network **(A)** power value curve of scale independence and mean connectivity for various soft thresholds (power). **(B)** Clustering dendrogram of all genes, dynamic tree cut and merged dynamics. Each color represents a gene co-expression module. **(C)** Heatmap of module-module membership. Red and blue represent positive and negative correlations, respectively. **(D)** Bar chart showing number of genes in each module.

The overall experimental workflow was illustrated in Supplementary Figure S1. After low-quality reads were filtered, an expression matrix consisting of 22 samples and 20,944 transcripts was used to construct the gene co-expression network. To meet the scale-free network, the soft thresholding power β = 6 (scale-free *R*^2^ = 0.9) was chosen for the present study. With this soft-threshold, the average connectivity was in line with the scale-free requirements (Figure 1A). In addition, according to the dynamic tree cut algorithm and merge cutoff of 0.75, a total of 16 gene co-expression modules were generated and assigned different colors for distinguishing each other (Figure 1B). The correlation heatmap of each module revealed that the majority of modules were independent of each other, except for the light green and sky blue modules (Figure 1C). Additionally, the number of genes in each module varied widely. For example, the light green module comprised 515 genes, the turquoise module contained the maximum number of genes, whereas the darkolivegreen module contained the minimum number of genes (Figure 1D).

### 3.2 Identification and functional enrichment analysis of the key modules associated with PAH irreversibility

**Figure 2:**
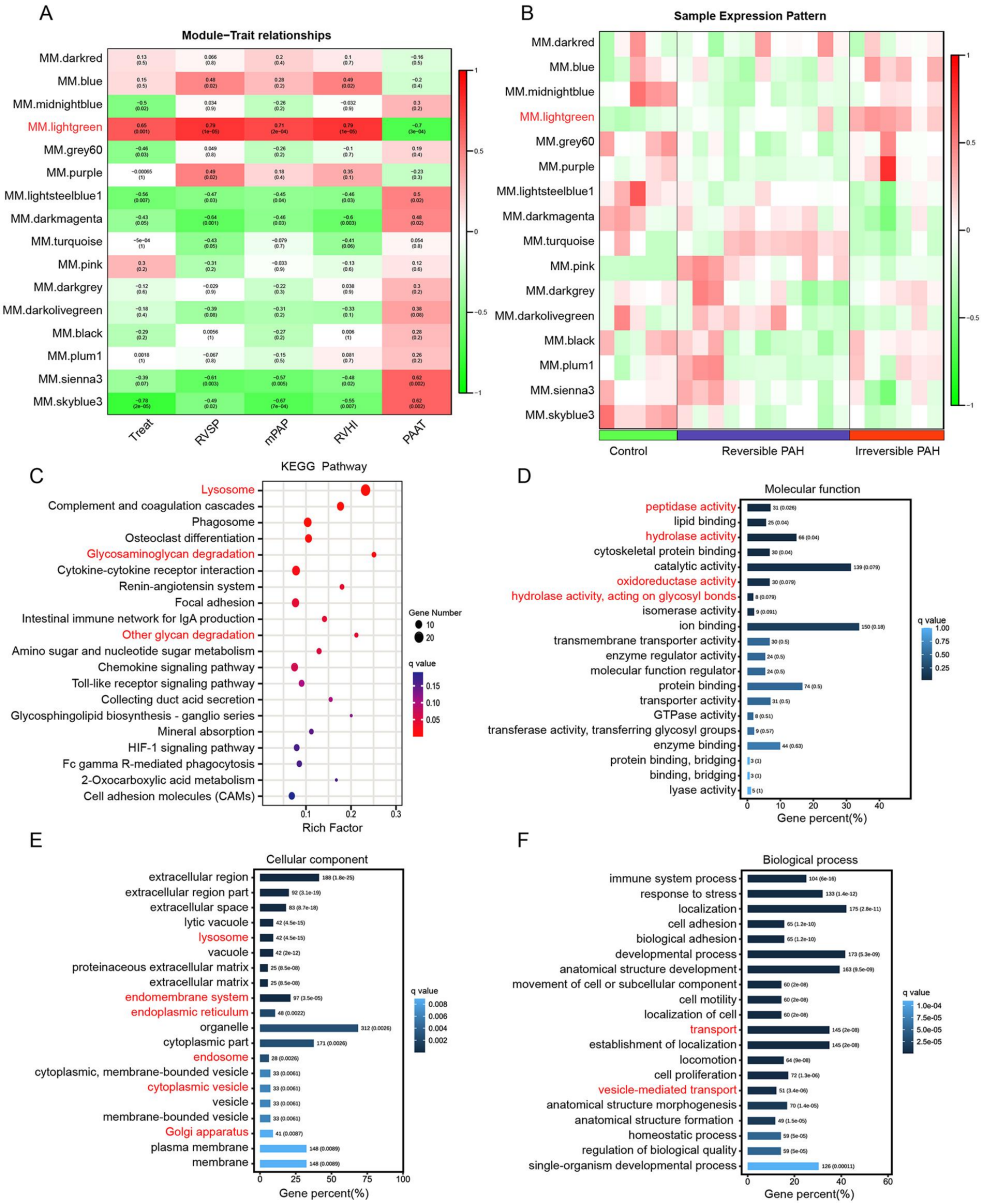
Identification of the key modules associated with PAH irreversibility and functional enrichment analysis **(A)** heatmap plot of module–trait relationships. The row and column represent co-expression modules and phenotypic traits, respectively. **(B)** Heatmap plot of the sample expression patterns. The row and column represent co-expression modules and sample types, respectively. **(C)** KEGG pathway enrichment analysis of the key module. The top 20 KEGG pathways are showed. **(D–F)** Gene Ontology enrichment analysis of the light green module. The top 20 GO terms in terms of molecular function **(D)**, cellular component **(E)** and biological process **(F)** are showed. A p-adj **(q)** -value <0.05 was considered to indicate significant enrichment.

The irreversibility of MCT + shunt- induced PAH was assessed by the phenotypic traits mPAP, RVHI, RVSP and PAAT (6). To identify the gene co-expression modules related to the irreversible PAH, we correlated co-expression modules with phenotypic traits through Pearson correlation analysis. As shown in Figure 2A, the light green module was highly positively correlated with RVSP (*r* = 0.79, *p* = 1e-05), RVHI (*r* = 0.79, *p* = 1 × 10^−05^) and mPAP (*r* = 0.71, *p* = 2 × 10^−04^) and negatively correlated with PAAT (*r* = −0.7, *P* = 3 × 10^−04^). In contrast to the light green module, the sky blue module showed an inverse correlation. Heatmap of the sample expression patterns revealed that the light green module was in accordance with the change of phenotypic traits (Figure 2B). Therefore, the light green module was most relevant to PAH irreversibility and was identified as the key module for functional analysis. KEGG pathway analysis revealed that the light green module was significantly enriched in the lysosome, glycosaminoglycan degradation and other glycan degradation terms (Figure 2C). GO enrichment analysis of the light green module revealed that enriched molecular functions were associated mainly with a variety of lysosomal enzyme catalytic activities, including peptidase activity, hydrolase activity and oxidoreductase activity (Figure 2D). The cellular component was associated with lysosome, and the intracellular endomembrane system responsible for lysosomal biogenesis, including endoplasmic reticulum, Golgi apparatus, cytoplasmic vesicle and endosome (Figure 2E). Biological processes were associated with intracellular transport, such as vesicle-mediated transport (Figure 2F). Collectively, functional enrichment analysis of key module indicated that the irreversibility of MCT + shunt-induced PAH was associated with lysosomal dysfunction.

### 3.3 Involvement of dysfunctional lysosome in irreversible PAH

Lysosomal biogenesis and autophagy are regulated by the transcription factor EB (TFEB) (14). 73 TFEB target genes play a well-known role in lysosomal function (15). To determine whether lysosome is dysfunctional in irreversible PAH, we analyzed the expression of 73 genes with a defining role in lysosomal function. Differential expression analysis using a threshold of p-adj ≤0.05 showed that 29 of the 73 TFEB target genes were differentially expressed in irreversible PAH, including 18 upregulated and 11 downregulated genes (Supplementary Figure S2A). Hierarchical clustering of the DEGs revealed elevated expression of lysosomal hydrolases including cathepsins (Ctsd, Ctsa and Ctsb), Glb1, ppt1, Galns and the lysosomal membrane proteins Glmp and Cd63 (Supplementary Figure S2B). Additionally, the expression of V-type ATPases (V-ATPases), which are multisubunit enzymes that mediate lysosomal acidification, was dysregulated, due to increased Atp6v1b2 and reduced Atp6v0b (Supplementary Figure S2C). In contrast, the expression of non-lysosomal proteins involved in lysosomal biogenesis, including M6pr, Igf2r and Nagpa, was reduced (Supplementary Figure S2D). M6pr and Igf2r are the mannose 6-phosphate (M6P) receptors responsible for the intracellular targeting of multiple lysosomal enzymes and a deficiency of M6pr and Igf2r results in the missorting of lysosomal enzymes (16). Like the expression of genes involved in lysosomal biogenesis, the expression of autophagic genes, including Vps26a, Uvrag and Vps33a was reduced in irreversible PAH (Supplementary Figure S2E). Therefore, TFEB-mediated lysosomal biogenesis and autophagy are impaired in irreversible PAH.

**Figure 3:**
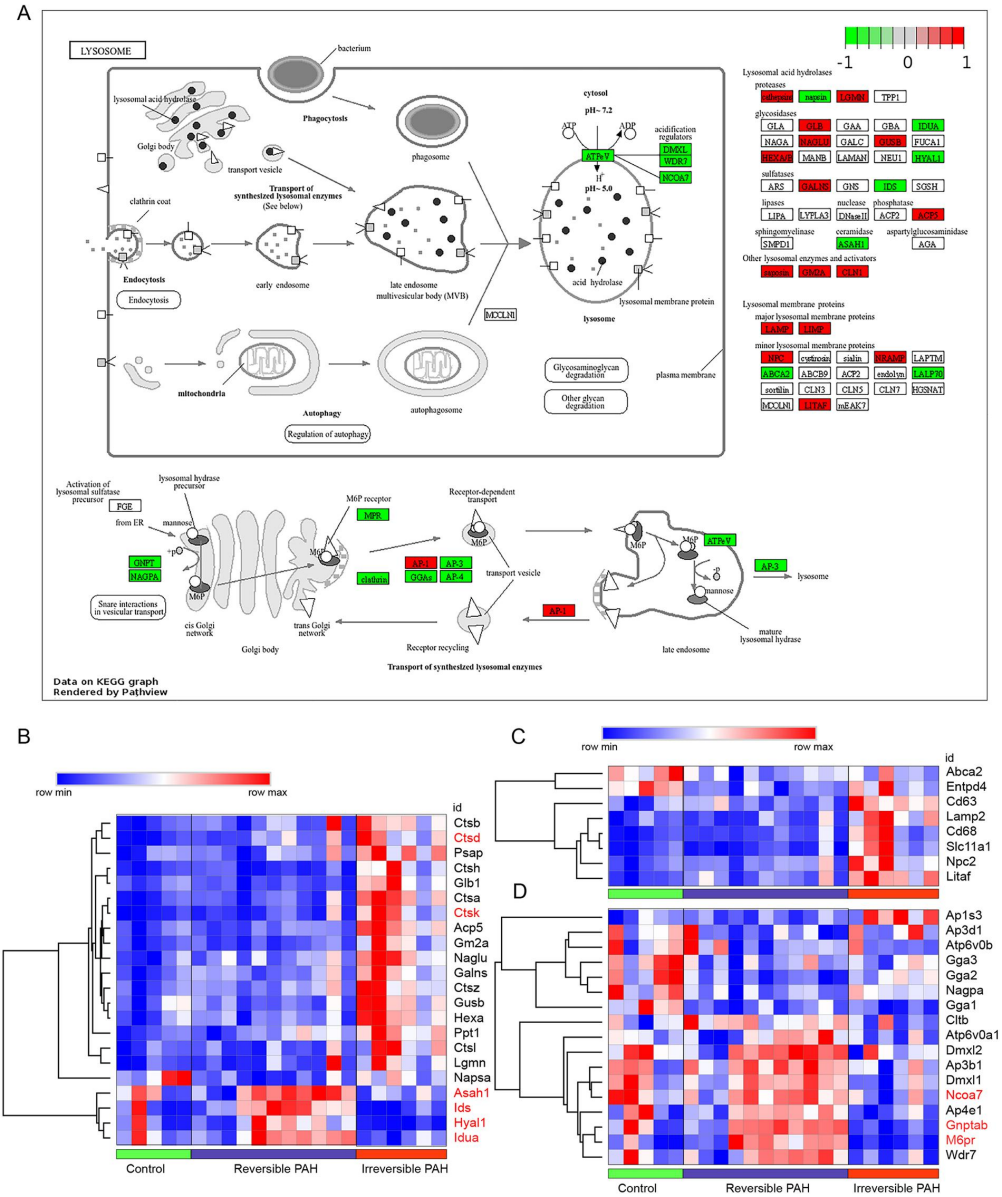
Involvement of dysfunctional lysosome in irreversible PAH **(A)** integration and visualization of gene expression changes in the lysosomal pathway using modified pathview. The color represents padj value for the comparison of irreversible PAH with the control. The genes with increased and reduced expression are shown in red and green, respectively. **(B–D)** Heatmap showing lysosomal enzymes **(B)**, lysosomal membrane proteins **(C)** and non-lysosomal proteins involved in lysosomal biogenesis **(D)** that were annotated in lysosome in the KEGG database. The rows in the heatmap represent gene expression levels, and columns represent each sample. Differentially expressed genes were identified by using a threshold of p-adj ≤0.05.

The KEGG results revealed that lysosome was the most significantly enriched pathway (Figure 2C). Pathview and hierarchical clustering analysis of lysosome showed that the dysregulated DEGs were associated with lysosomal enzymes, membrane proteins and proteins involved in lysosomal biogenesis and acidification in irreversible PAH (Figures 3A–D). As shown in Figure 3B, lysosomal enzymes exhibited dysregulated expression in irreversible PAH, including the upregulation of lysosomal proteases (Ctsd, Ctsk, Ctsl, Ctsz and Lgmn), glycosidases (Glb1, Naglu and Gusb), sulfatase (Galns), phosphatase (Acp5), and other lysosomal enzymes and activators (Psap, Gm2a and Ppt1), as well as the downregulation of proteases (Napsa), glycosidases (Idua and Hyal1), sulfatase (Ids) and acid ceramidase (Asah1). Among these lysosomal enzymes, Lgmn (17), Cathepsins [Ctsd (3), Ctsl (18), and Ctsz (19)] and reduced Asah1 (20) have been reported to be associated with pulmonary hypertension. The dysregulated results were also showed in the lysosomal membrane proteins, because of upregulation of Lamp2, Cd68, Cd63 and Npc2, and downregulation of Abca2 and Entpd4 (Figure 3C). Interestingly, except for Ap1s3 involving vesicular bidirectional transport between the Golgi complex and endosome (21), all proteins involved in transport of synthesized lysosomal enzymes to the lysosome were downregulated in irreversible PAH (Figure 3D), including Gnptab, M6pr, Nagpa, Cltb, monomeric Golgi-localized, ear-containing, Arf-binding proteins GGAs (Gga1, Gga2 and Gga3) and subunits of cargo adaptor protein (AP) complex (Ap3b1, Ap3d1 and Ap4e1). Lysosomal enzyme transport requires cargo adaptors AP complexes and GGAs (21). The identification of downregulated GGAs and AP complexes indicated impaired transport of newly synthesized lysosomal enzymes. Similar results were shown for the lysosomal acidification due to the reduced expression of lysosomal acidification and regulators, including Dmxl1, Dmxl2, Wdr7 and Ncoa7 (Figure 3D). Interestingly, a recent study revealed that an endothelial Ncoa7 deficiency resulted in lysosomal dysfunction and worsened PAH (22). These results indicated the impairment of lysosomal transport and acidification in irreversible PAH.

### 3.4 Identification of differentially expressed genes associated with lysosome

**Figure 4:**
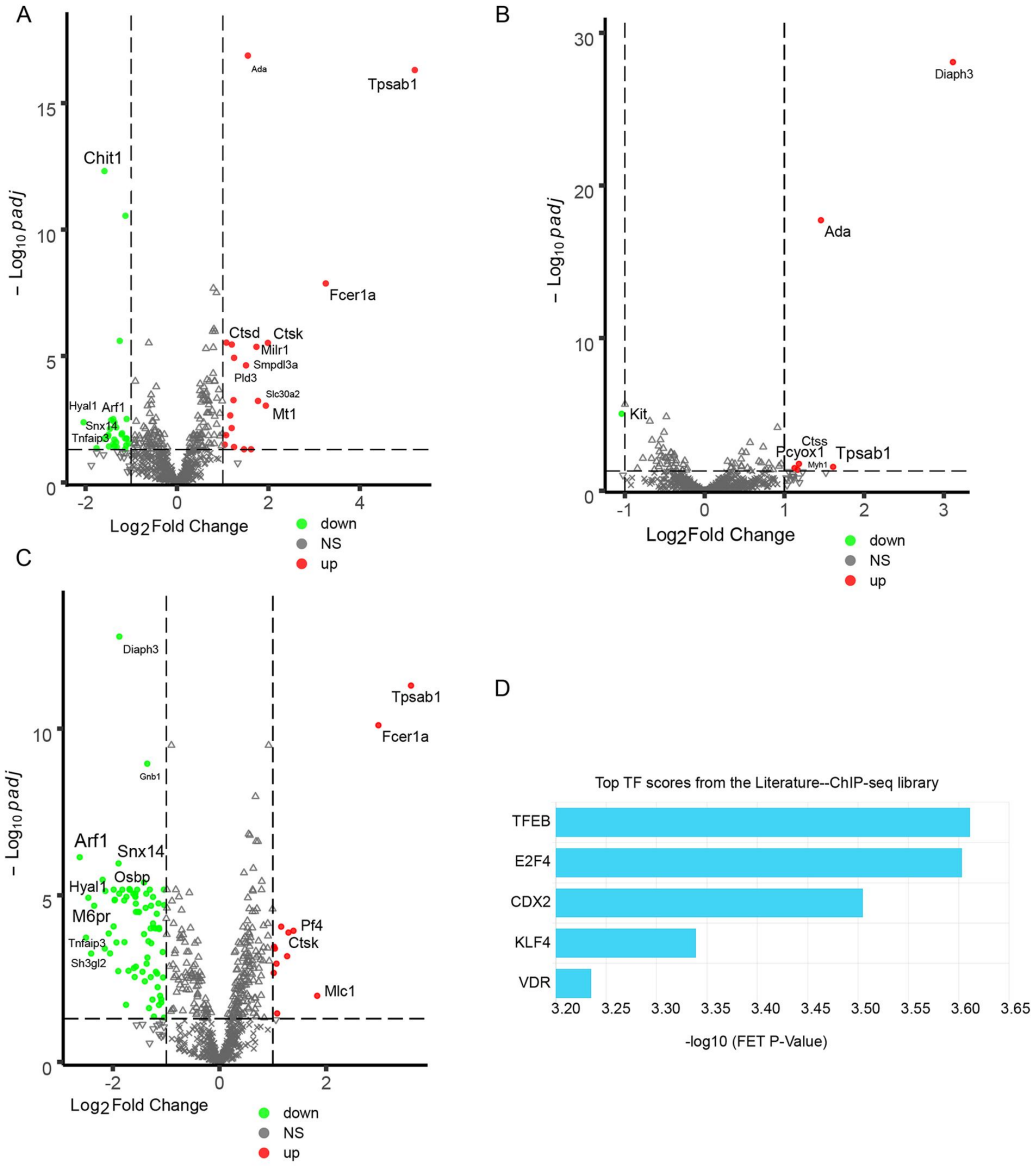
DEGs associated with lysosome and transcription factor enrichment analysis. **(A–C)** Volcano plot showing DEGs in the comparison of irreversible PAH with the control **(A)**, reversible PAH with the control **(B)** and irreversible PAH with reversible PAH **(C)**. The top 20 DEGs are shown in each figure. **(D)** Enrichment of highly represented transcription factors among the reduced DEGs by using ChEA3 database. Only the top 5 transcription factors are shown. The significantly enriched terms were determined by the top TF scores from the literature-ChIP-seq library.

To comprehensively characterize dysfunctional lysosome in PAH, we collected genes associated with lysosome from databases of hLGDB (13), GO and KEGG and via literature review, and a total of 819 genes were collected (Supplementary Table S1). Differentially expressed genes (DEGs) were identified by using a more stringent threshold of log2Fold Change ≥ 1 and p-adj ≤0.05. In total, 113 of the 819 genes were differentially expressed. For displaying DEGs, a volcano plot was generated in each comparison. Volcano plot showed the top- upregulated DEGs Tpsab1, Fcer1a, Ctsk and Pcyox1, as well as the top- downregulated DEGs Arf1, M6pr, Hyal1 and Chit1 (Figures 4A–C). Hierarchical clustering analysis of all DEGs revealed the downregulation of numerous genes related to lysosome, including DEGs involved in lysosomal trafficking (Gnptab, M6pr and Lyset), small GTP-binding protein Rab family members (Rab5a, Rab12 and Rab7a), SNAREs (Vamp8, Stx7 and Vti1b) and Snap23 (Supplementary Figure S3). A recent report showed that Rab7 was reduced in PAH, leading to impaired endolysosomal trafficking (23). Gnptab is a regulator of M6P-dependent Golgi-to-lysosome trafficking of lysosomal enzymes (24), and genetic mutation of Gnptab resultes in mucolipidosis II complicated by severe pulmonary hypertension (25). Enrichment analysis of the reduced DEGs using ChEA3 revealed that TFEB, a transcription factor linked to autophagy and lysosomal biogenesis (14), was the most significantly enriched transcription factor (Figure 4D). In addition to the 6 sustained downregulated DEGs, 85 reduced genes showed a dichotomous expression pattern clearly distinguishing irreversible PAH from reversible PAH (Supplementary Figure S3), which coincided with a switch from reversible to irreversible PAH vascular phenotype.

### 3.5 Functional enrichment analysis of the reduced DEGs that present a switch from reversible to irreversible PAH

**Figure 5:**
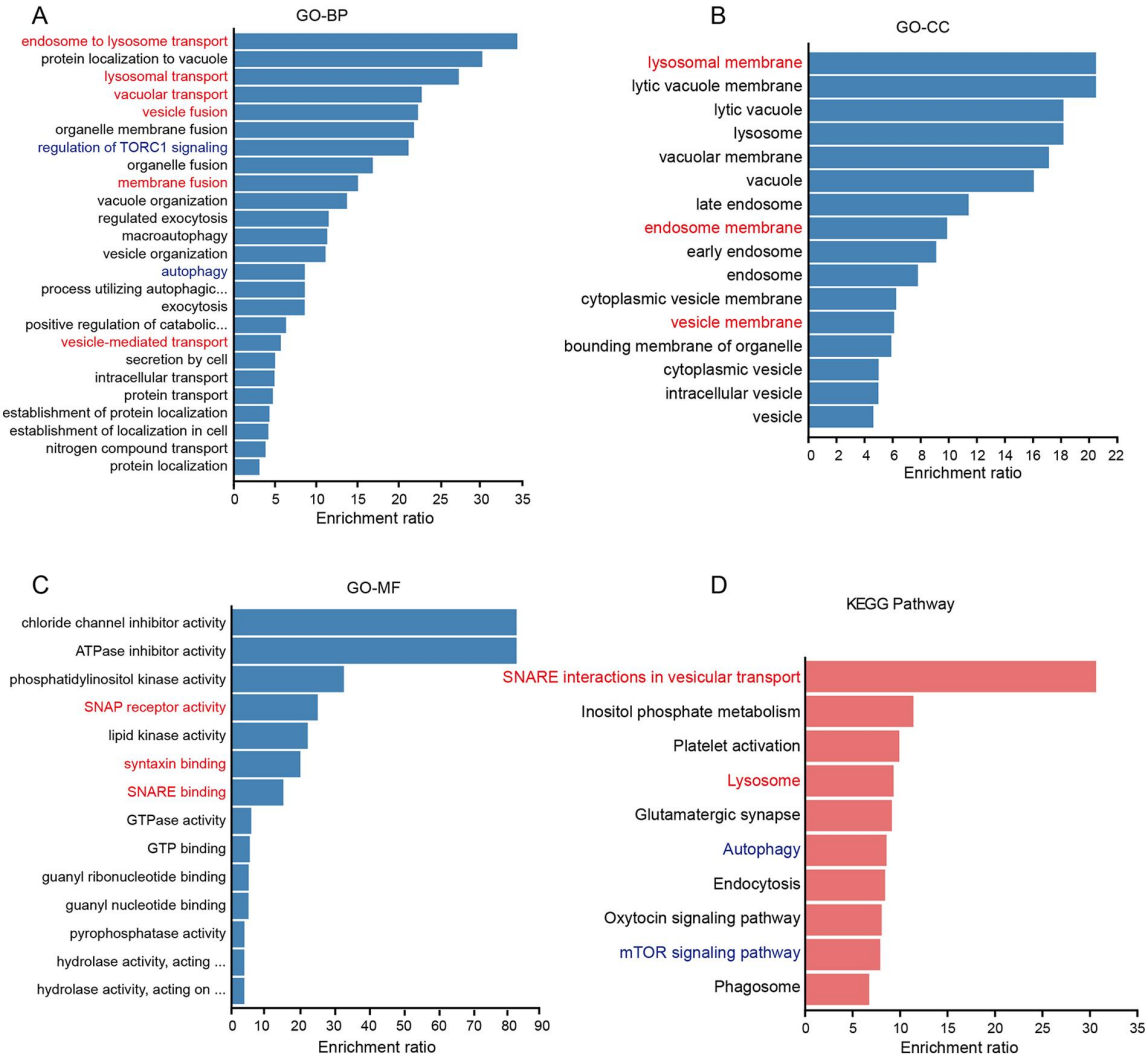
Functional enrichment analysis of the reduced DEGs with a switch from reversible to irreversible PAH. **(A–C)** Gene Ontology enrichment analysis of the reduced DEGs. The significantly enriched GO terms of biological process **(A)**, cellular component **(B)** and molecular function **(C)** are shown. **(D)** KEGG pathway enrichment analysis of the reduced DEGs. A padj value <0.05 was viewed as significant enrichment.

We focused on 85 reduced DEGs whose expression patterns presented a switch from reversible to irreversible PAH. GO enrichment analysis revealed that this set of reduced DEGs was associated mainly with the biological processes of the intracellular transport of lysosomal proteins and autophagy, such as endosome to lysosome transport, vesicle-mediated transport and vesicle fusion (Figure 5A). The cell component of the reduced DEGs was the endomembrane associated with intracellular transport, including vesicle membrane, endosome membrane and lysosomal membrane (Figure 5B). The molecular function of the reduced DEGs was associated with membrane fusion, including SNARE binding, syntaxin binding and SNAP receptor activity (Figure 5C). KEGG pathway enrichment of the reduced DEGs showed 10 significantly enriched pathways, among which 4 pathways such as SNARE interactions in vesicular transport, autophagy and lysosome, were overlapped in GO analysis results (Figure 5D). Overall, the reduced DEGs exhibited a switch from reversible to irreversible PAH were associated with lysosomal trafficking and sorting. According to functional enrichment results, 56 of the 85 reduced DEGs were annotated in lysosomal trafficking and sorting and likewise, exhibited a switch from a reversible to irreversible PAH, such as Gnptab, M6pr, Arf1, Ids and SNAP23 (Supplementary Figure S4A). Based on our dataset GSE149713, 36 of the 56 (64.3%) DEGs were confirmed to be reduced in the initiation and progression of MCT-induced PAH, including Gnptab, M6pr, Ids, Tsc1, Arf1, Rnf13, Zfyve16 and Snap23 (Supplementary Figure S4B).

### 3.6 Validation of DEGs related to lysosomal trafficking and sorting in CHD patients with irreversible PAH

To determine whether lysosomal enzyme trafficking and sorting are impaired in CHD patients with irreversible PAH, we analyzed dataset GSE254617 and investigated the expression of 56 genes, in combination with key genes involving transport of synthesized lysosomal enzymes in KEGG pathway, including ARF small G proteins, cargo adaptors of AP complexes and GGAs, Rab GTPases, SNAREs (26) and coat protein clathrin. A total of 32 genes were differentially expressed, including 11 upregulated and 21 downregulated genes (Supplementary Figure S5A). The downregulated genes included M6PR, AP3B1, AP3D1, ARF1, ARF4, CLTC, RAB5A, STX7, SNAP23 and NSF in CHD patients with irreversible PAH (Supplementary Figure S5B).

**Figure 6:**
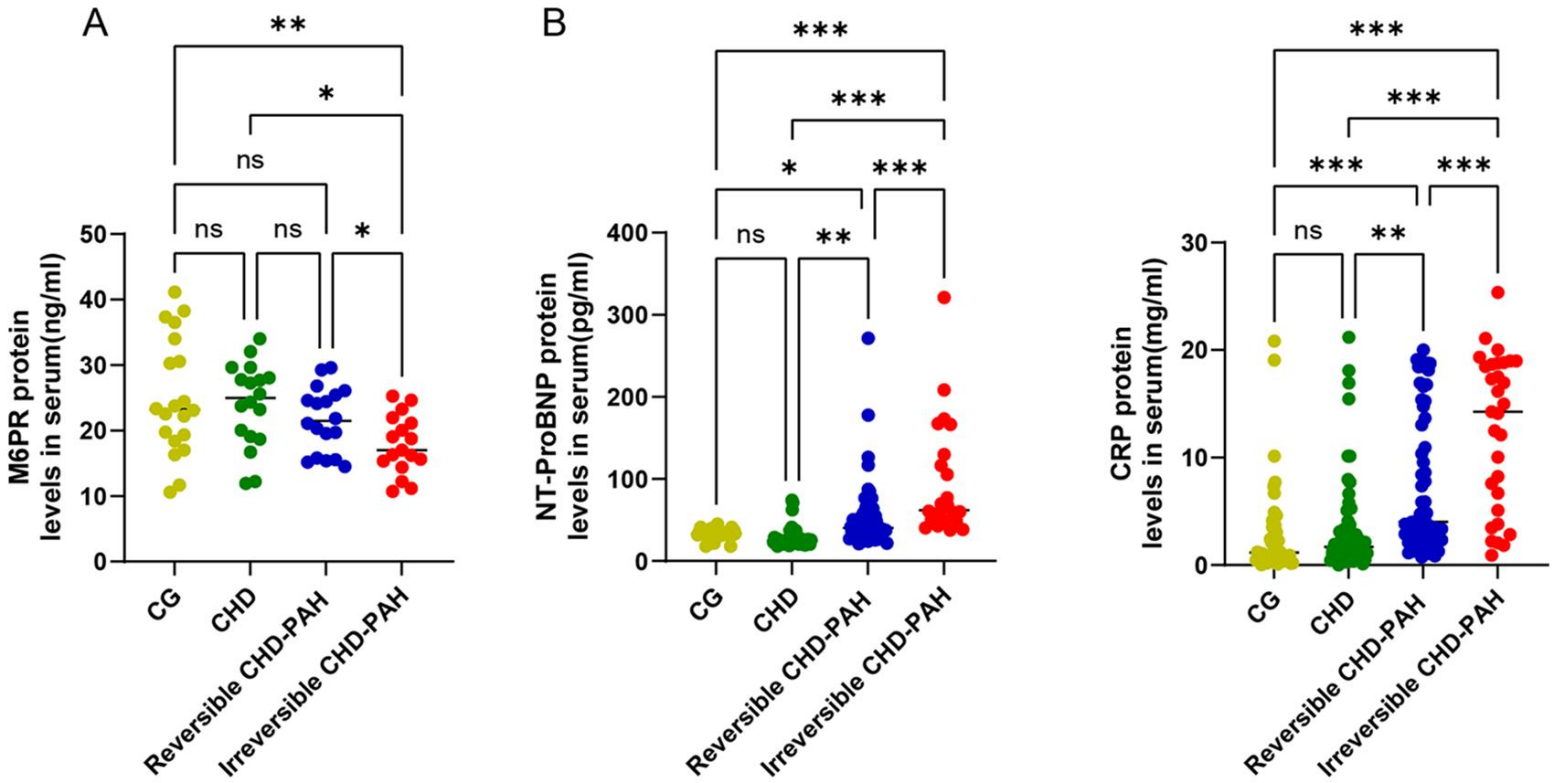
Validation of M6PR protein levels in serum. **(A)** Serum levels of M6PR in CG, CHD, reversible PAH, and irreversible PAH. **(B)** Serum levels of NT-proBNP and CRP in CG, CHD, reversible PAH, and irreversible PAH. CG, control group; CHD, congenital heart disease; CHD-PAH, congenital heart disease-associated pulmonary arterial hypertension. *p < 0.05, **p < 0.01, and ***p < 0.001.

Notably, M6PR plays a critical role in lysosomal trafficking and sorting by mediating the specific transport of M6P-tagged acid hydrolases from the Golgi apparatus or the plasma membrane to lysosomes. M6PR is secreted into the peripheral circulation via exosomes (27), therefore, we assessed serum M6PR levels in patients with CHD-PAH. This study included 40 CHD-PAH patients with 20 cases in the reversible group and 20 cases in the irreversible group. Baseline demographic and clinical characteristics are presented in Supplementary Table S2. No significant differences were observed between the two groups with respect to sex distribution and age. Hemodynamic assessments revealed significantly elevated values in the irreversible group compared with the reversible group, including mPAP, RVSP, and PVR. ELISA analysis revealed significantly decreased M6PR protein levels in patients with irreversible CHD-PAH, in contrast to the well established biomarkers NT-proBNP and CRP (Figure 6). Moreover, serum M6PR concentrations were markedly lower in patients with irreversible PAH than in those with reversible CHD-PAH (Figure 6A), indicating impaired M6P-dependent lysosomal trafficking in irreversible PAH.

### 3.7 Identification and validation of lysosomal hub genes associated with PAH irreversibility

According to the thresholds |MM|≥ 0.8 and |GS|≥ 0.5, a total of 102 candidate hub genes were identified in the light green module, 62 of which were differentially expressed (Supplementary Figures S6A, C). Thus, these genes were considered hub genes. 8 of the 62 hub genes were related to lysosome and the expression of 8 hub genes, including Ctsd, Ctsk, Ctsb, Ctsa, Pld3, Tpsab1, Lgals3 and Smpdl3a, was in line with changes in the phenotypic traits used for assessment of PAH irreversibility (Supplementary Figures S6B, C). Additionally, validation of 8 hub genes using our dataset GSE149713 revealed increased expression in the lung tissue during the progression of MCT-induced PAH (Supplementary Figure S6D).

**Figure 7:**
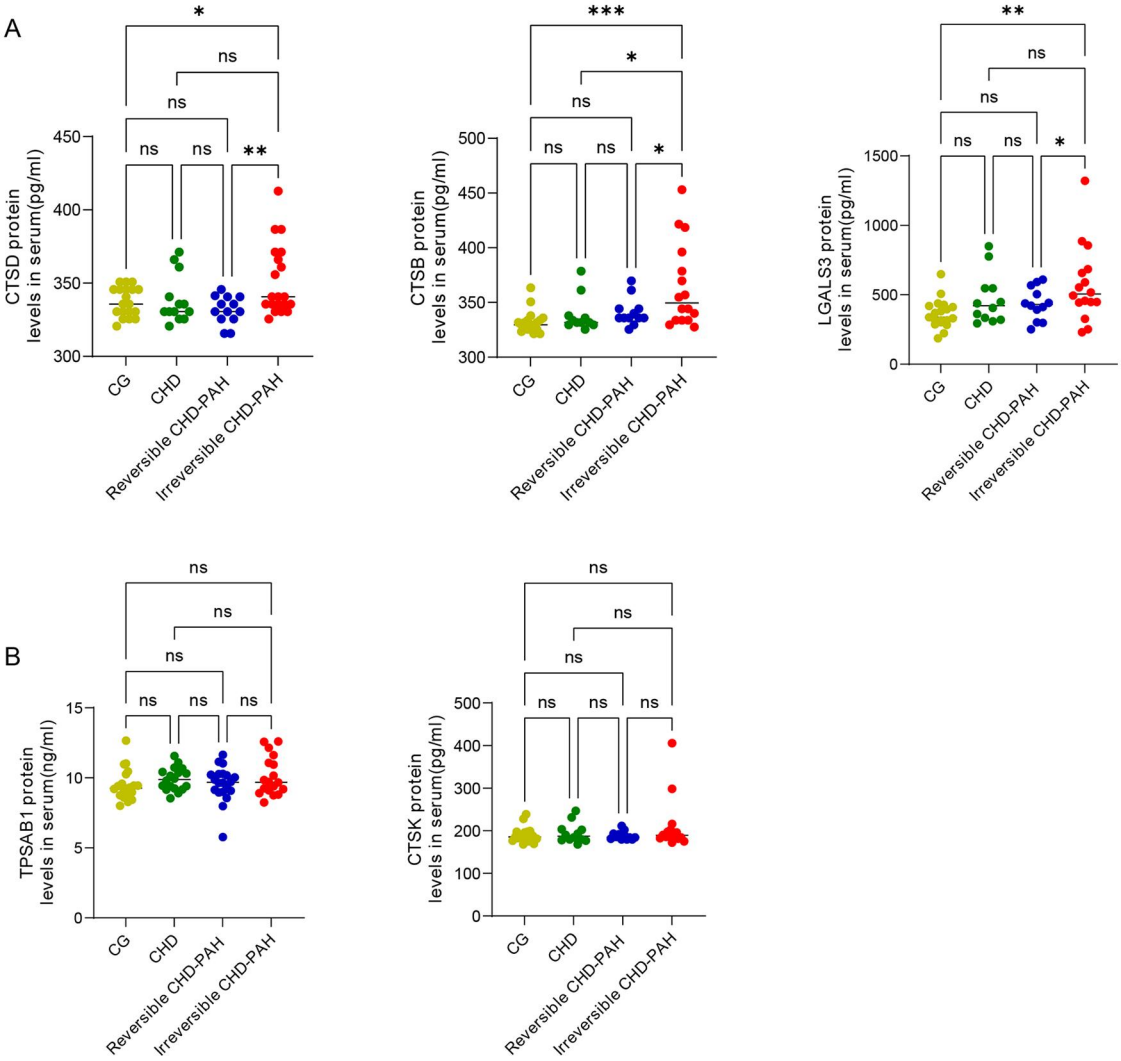
Protein levels of lysosomal hub genes in serum. **(A)** Serum levels of CTSD, CTSB and LGALS3 in CG, CHD, reversible PAH, and irreversible PAH. **(B)** Serum levels of lysosomal TPSAB1 and CTSK in CG, CHD, reversible PAH, and irreversible PAH. CG, control group; CHD, congenital heart disease; CHD-PAH, congenital heart disease-associated pulmonary arterial hypertension. *p < 0.05, **p < 0.01, and ***p < 0.001.

M6PR is required for M6P-mediated trafficking and sorting of lysosomal enzymes. In the absence of M6PR, lysosomal enzymes are secreted into the extracellular space (such as serum and urine) rather than being directed to lysosomes (28). As a result, we investigated whether serum levels of the lysosomal enzymes CTSD, CTSK, CTSB, and TPSAB1 were elevated in patients with irreversible CHD-PAH. ELISA analyses revealed significantly elevated serum levels of CTSD, and CTSB in irreversible PAH (Figure 7A), whereas CTSK, and TPSAB1 levels did not show a significant change (Figure 7B). In addition, we assessed serum levels of LGALS3, a lysosomal damage biomarker (29), in patients with irreversible CHD-PAH. ELISA results demonstrated markedly elevated LGALS3 protein levels in irreversible PAH (Figure 7A). Compared to those with reversible PAH, patients with irreversible PAH exhibited significantly higher levels of CTSD, CTSB, and LGALS3 in the serum (Figure 7A). Notably, there were no significant differences in levels of CTSD, CTSB, and LGALS3 among reversible CHD-PAH, CHD only, and healthy controls, contrasting sharply with the established biomarkers NT-proBNP and CRP (Figure 6B). Collectively, these findings support a defect in M6P-dependent lysosomal enzyme trafficking and sorting in irreversible PAH.

### 3.8 Effect of M6PR knockdown on lysosomal enzyme secretion, proliferation and migration of PASMCs

**Figure 8:**
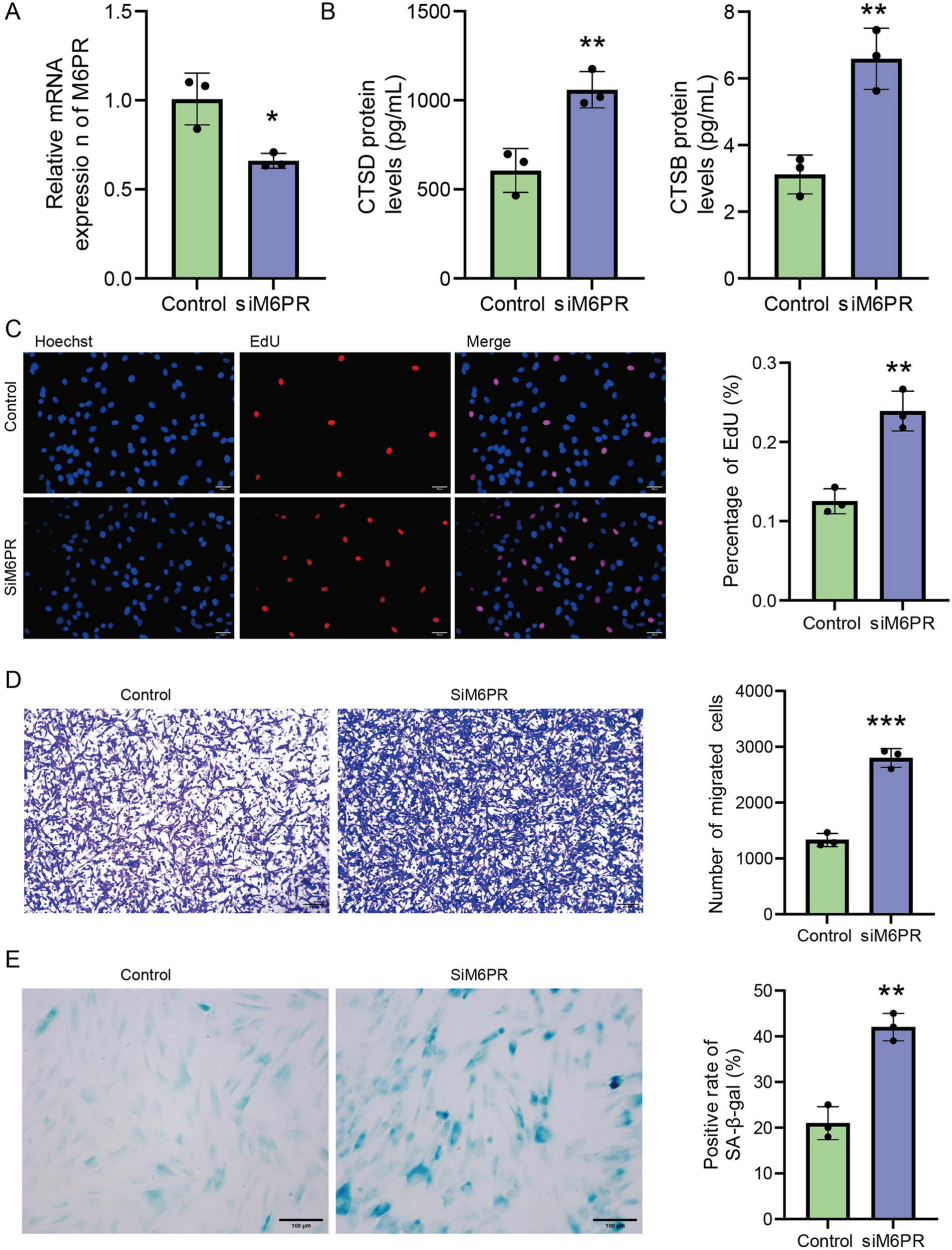
Effect of M6PR knockdown on lysosomal enzyme secretion, proliferation, and migration in PASMCs **(A)** RT-qPCR analysis of M6PR mRNA expression following transfection with siM6PR. **(B)** Serum levels of CTSD and CTSB in the cell culture supernatant upon M6PR depletion. **(C)** EdU incorporation assay demonstrating significantly enhanced PASMC proliferation upon M6PR knockdown. **(D)** Transwell migration assay revealing increased migratory capacity of PASMCs following M6PR silencing. **(E)** SA-β-gal staining indicating reduced cellular senescence in M6PR-deficient PASMCs. *p < 0.05, **p < 0.01, and ***p < 0.001.

We used siRNA to specifically knock down M6PR in rat PASMCs. RT-qPCR analysis confirmed a significant reduction in M6PR mRNA expression following transfection with siM6PR (Figure 8A). Consistent with impaired lysosomal trafficking and sorting, ELISA results revealed markedly increased levels of CTSD and CTSB in the cell culture supernatant upon M6PR depletion (Figure 8B). EdU incorporation assays revealed that M6PR knockdown markedly enhanced PASMC proliferation (Figure 8C). Similarly, Transwell migration assay demonstrated M6PR deficiency significantly increased migratory capacity of PASMCs (Figure 8D). Given prior evidence that M6PR upregulation mitigates cellular senescence (30), we assessed senescence status via Senescence-Associated β-Galactosidase (SA-β-gal) staining, a well-established histochemical marker of cellular senescence (31). As shown in Figure 8E, M6PR knockdown led to a pronounced increase in SA-β-gal–positive PASMCs.

## 4 Discussion

The lysosome is a membrane separating organelle in animal cells, which acts as the main digestive compartment of cell. The lysosome contains more than 40 hydrolases in an acidic environment and various macromolecules are transported to it for degradation. Autophagy is an evolutionarily conserved lysosome- dependent catabolic process and dysfunctional autophagy has been well reported in PAH (32). All three autophagic processes, macroautophagy, microautophagy and chaperone-mediated autophagy, culminate in lysosomal degradation. However, dysfunctional lysosome has been somewhat overlooked in PAH. In the present study, WGCNA was used to identify modules associated with PAH irreversibility. We found that the light green module was associated with the phenotypic traits of PAH irreversibility. Functional enrichment and Pathview analysis revealed dysfunctional lysosome in irreversible PAH and moreover, PAH irreversibility was associated with a switch from compensated to decompensated lysosomal trafficking and sorting.

Increased markers of cellular senescence have been demonstrated in PAH (33), and cell senescence contributes to the development of irreversible PAH (6). Cell senescence and lysosomal dysfunction are highly interconnected. On the one hand, one of most defining hallmarks of a senescent cell is an increase in lysosomal content, which is measured by senescence-associated beta-galactosidase (SA-β-gal) (34, 35). In fact, SA-β-gal is a lysosomal beta-galactosidase (Glb1) (36). Senescence-Associated β-Galactosidase (SA-β-gal) staining is a well-established histochemical marker of cellular senescence (31). Cellular senescence was assessed by SA-β-gal staining, SA-β-gal staining showed that M6PR knockdown resulted in a pronounced increase in SA-β-gal–positive PASMCs. Similarly, Ctsd, a lysosomal protein, is also enriched in senescent cells and is used as a biomarker of cellular senescence (37). In the present study, the expression of cellular senescence biomarkers SA-β-gal (Glb1) and Ctsd was increased in both mRNA and protein levels, which was consistent with the role of cellular senescence in irreversible PAH (6). On the other hand, the lysosomal proteome of senescent cells contributes to the senescence secretome (38), and moreover, TFEB-dependent lysosome biogenesis is required for senescence (34). Given that cellular senescence impairs PAH reversibility (6), it is possible that lysosomal dysfunction contributes to cellular senescence and the consequent development of irreversible PAH.

A defining feature of lysosomes is their ability to maintain an acidic pH of approximately 4.5, a critical condition for the optimal activity of hydrolytic enzymes. Lysosomal acidification is controlled by V-ATPases protein complexes encoded by multiple ATP6 genes (39). The identification of downregulated subunits of V-ATPases Atp6v0a1 and Atp6v0b, as well as upregulated subunits Atp6v1b2, Atp6v0d2, Atp6v1c1 and Atp6v1d indicated dysregulated lysosomal acidification in irreversible PAH. Of V-ATPase subunits, Atp6v1b2, an Ncoa7 binding partner, was reported to be upregulated in PAH. In addition to dysregulated V-ATPases, lysosomal acidification regulators, including Ncoa7, Dmxl1, Dmxl2 and Wdr7, presented increased and reduced expression, which is consistent with a switch from a reversible to irreversible PAH. Ncoa7 binds to V-ATPases to control lysosomal acidification. Interestingly, a recent study showed that Ncoa7 deficiency promoted lysosomal dysfunction and that mice deficient in Ncoa7 exhibited worsened endothelial cell immunoactivation and more severe PAH (22). Furthermore, the SNP rs11154337 in Ncoa7 regulated its expression, and lysosomal acidification, and was correlated with PAH severity and mortality in two cohorts of PAH patients (22).

Lysosomal enzymes are synthesized in the endoplasmic reticulum and transferred to the Golgi apparatus, where the precursor proteins are modified with M6P residues. The M6P residue is a lysosomal targeting signal, allowing recognition of lysosomal enzymes by M6P receptors (MPRs) (24). The M6P marker is generated through a two-step reaction catalyzed by two Golgi enzymes, Gnptab and Nagpa (40). First, Gnptab transfers N-acetylglucosaminel-phosphate to select mannose residues on lysosomal enzymes, forming a phosphodiester intermediate. The enzyme Nagpa subsequently removes the N-acetylglucosamine, exposing the M6P monoester signal. After the generation of M6P residue, lysosomal enzymes bind to M6P-specific receptors (M6pr and Igf2r) in the Golgi, facilitating their segregation from proteins destined for secretion. The ligand‒receptor complex subsequently exits the Golgi via clathrin-coated vesicles and is delivered to endosome and lysosome (40). Without M6P residues, lysosomal enzymes are secreted into the extracellular space instead of being transported to the lysosome. Notably, we found a remarkable reduction in the expression of Gnptab, Nagpa and M6pr in irreversible PAH, of which Gnptab and M6pr presented a distinct expression pattern that clearly distinguished irreversible PAH from reversible PAH. Mutations in the Gnptab gene encoding the catalytic subunits of GlcNAc-1-phosphotransferase enzyme, result in the mucolipidosis type II (40). Mucolipidosis type II, an inherited lysosomal storage disease, is characterized by impaired enzyme transport into the lysosome and leakage into the extracellular space (40). Interestingly, patients with mucolipidosis type II are complicated with severe PAH and autophagic dysfunction (25, 41–43). Moreover, PAH-targeted therapy is effective and leads to a reduction in right ventricular systolic pressure (25). Given the role and particular expression pattern of Gnptab and M6pr, as well as elevated protein levels of lysosomal enzymes CTSD and CTSB in the serum, the loss of M6P-mediated lysosomal trafficking and sorting was likely associated with a transition from reversible to irreversible PAH.

The typical diseases of lysosomal dysfunction are lysosomal storage disorders (LSDs). LSDs are a group of rare inherited disorders characterized by defects in lysosomal function, resulting in the accumulation of undegraded materials within lysosome (44). The causes of LSDs include deficiency of lysosomal enzymes and membrane proteins, and defects in enzyme transport into the lysosome (44). Notably, patients with LSDs often develop pulmonary vascular remodeling and pulmonary hypertension, and examples of such LSDs include mucopolysaccharidoses (45–47), Gaucher disease (48), Pompe disease (49) and mucolipidoses (25, 41). Mucopolysaccharides, also known as glycosaminoglycans, include heparan sulfate, dermatan sulfate, keratan sulfate, chondroitin sulfate and hyaluronic acid. Mucopolysaccharidoses (MPSs) are a group of LSDs caused by mutations in genes encoding lysosomal enzymes that degrade glycosaminoglycans. Pulmonary hypertension is a frequent findings in a variety of MPSs (45–47), including MPS type I (50, 51), MPS type II (52, 53), MPS type IVA (54), MPS type VI (55, 56) and MPS type VII (57). Taking MPS typeⅠandⅡ as examples, MPS type I, historically known as Hurler's syndrome, is caused by a mutation in the Idua gene, leading to deficient alpha-L-iduronidase activity. Severe pulmonary hypertension with cardiopulmonary diseases was found in patients with MPS type I, and enzyme replacement therapy with laronidase reduced the elevated mPAP (51, 58). MPS type II is the most common type of mucopolysaccharidoses (59). MPS type II is caused by a mutation in Ids encoding iduronate 2-sulfatase which degrades heparan sulfate and dermatan sulfate (52). Pulmonary hypertension has been reported in patients with MPS type II and is one of the causes of death (52, 53). In the present study, the expression of Idua and Ids was reduced in irreversible PAH and exhibited an expression pattern distinguished irreversible PAH from reversible PAH, indicating a role of Idua and Ids in the development of irreversible PAH. Gaucher disease, caused by the mutations in gene encoding glucocerebrosidase, is the most common LSD (60). The development of PAH in patients with Gaucher disease has been well-characterized, and PAH-specific treatments were beneficial for patients with Gaucher disease-associated PAH, including reduced mPAP, increased exercise capacity and improved right heart failure (48, 61–63). Additionally, histological examination of explanted lungs from patients with Gaucher disease revealed pulmonary vascular remodeling including medial hypertrophy, intimal fibrosis and plexogenic arteriopathy, a histopathological hallmark feature of PAH (64, 65). The pathological changes and response to PAH-specific therapy in patients with Gaucher disease are typical for that seen in patients with WHO group 1 PAH, suggesting that this condition should be reclassified as WHO Group 1 rather than Group 5 pulmonary hypertension (64).

Ceramide has been proposed to be a mediator of replicative senescence and has been shown to induce the expression of SA-β-gal in fibroblasts (66, 67). Acid ceramidase (Asah1) is the lysosomal hydrolase responsible for catalyzing the hydrolysis of ceramide into sphingosine and free fatty acid. Mutations in the Asah1 gene are causally linked to LSDs, such as Farber disease. Similar to Idua and Ids, expression of Asah1 was reduced in irreversible PAH and exhibited an expression pattern distinguished irreversible PAH from reversible PAH. Notably, acid ceramidase gene therapy ameliorates PAH with right heart dysfunction via decrease of pro-inflammatory factors, interleukins, and senescence markers (20).

The MPRs, M6pr and Igf2r, bind M6P-decorated lysosomal enzymes in the trans-Golgi network (TGN), leading to their package into clathrin-coated vesicles for transport to endosomes (24). The identification of reduced M6pr and Igf2r in irreversible PAH indicated a deficiency of newly synthesized enzyme binding at the TGN. The machinery of MPR trafficking from the TGN to endosomes comprises Arf small G proteins (such as Arf1 and Arf4), cargo adaptor protein (AP) complexes (AP-1, AP-3 and AP-4), Arf-binding proteins (Gga1, Gga2 and Gga3) and coat protein clathrin (Clta, Cltb and Cltc) (21). Notably, the Arf small G protein Arf1, a top reduced genes, regulates the formation of clathrin-coated vesicles at the TGN by promoting the recruitment of adaptor-protein complexes AP-1, AP-3 and AP-4, and the GGAs from the cytosol onto membranes (68). The expression of Arf1, subunits of the adaptor proteins Ap3b1, Ap3d1 and Ap4e1, the Arf-binding proteins Gga1, Gga2 and Gga3, and the subunits of clathrin Cltb and Cltc, was reduced in irreversible PAH. In addition, patients with irreversible PAH exhibited significantly higher levels of lysosomal enzymes CTSD and CTSB in the serum, compared to those with reversible PAH. Collectively, these results indicated the impairment of M6P-mediated trafficking machinery in irreversible PAH.

Lysosomal proteins are packaged into clathrin-coated vesicles and then transfer to the early endosome directly or through a constitutive secretory pathway via the plasma membrane. Lysosomes receive these enzymes when late endosomal–lysosomal fusion occurs. The progression from early to late endosomes is associated with Rab conversion involving Rab5 and Rab7 (69). The expression of Rab5a and Rab7a was reduced in irreversible PAH, indicating impaired late endosome formation. The fusion of late endosomes with lysosomes involves three sequential steps: tethering, the formation of trans-SNARE (SNAP receptor) complexes and membrane fusion (26). NSF, SNAPs and RAB7, play roles in tethering endosomes and lysosomes (70). In the present study, the expression of NSF and Rab7a was reduced. A recent study showed that Rab7 deficiency impairs endolysosomal trafficking and promotes pulmonary hypertension (23). The fusion of late endosomes and lysosomes requires the formation of SNARE complexes comprising Stx7, Stx8, Vti1b, Vamp7 or Vamp8 (26), of which Vamp8, Stx7 and Vti1b were found to be reduced in irreversible PAH. As a result, endosomal–lysosomal fusion is disrupted in irreversible PAH, possibly leading to impaired endosomal–lysosomal trafficking. The impairment of intracellular vesicular trafficking has been reported in a previous study showing that expression of the vesicular transport proteins NSF, *α*-SNAP and SNAP23 was reduced after PAH was established (71). Although disrupted endosomal–lysosomal fusion has not been reported directly, the disruption of endosomal-plasma membrane fusion has been intensively studied in the pathogenesis of PAH (72, 73). In MCT-induced PAH, disrupted intracellular membrane trafficking occurs mainly at the level of vesicle tethers, SNAREs and SNAPs (74). Previous sequential studies revealed that trapping of Golgi tethers, SNAREs and SNAPs in the Golgi membranes prevented trafficking of cargo proteins such as cav-1, eNOS and BMPR2 mutant to the plasma membrane, resulting in the development of PAH (72, 73). Of note, a similar machinery of vesicle tethers, SNAREs and SNAPs also mediates endosomal–lysosomal fusion during lysosomal enzyme trafficking (26).

In the present study, 8 hub genes were identified, including Lgals3, Ctsd, Ctsk, Ctsa, Ctsb, Tpsab1, Pld3 and Smpdl3a. Lgals3, a β-galactoside-binding cytosolic lectin, is frequently used to monitor lysosomal damage (29). Lgals3 is also identified as a biomarker for predicting CHD outcome after corrective surgery (75). Four cathepsins Ctsd, Ctsk, Ctsa and Ctsb were identified as hub genes, of which Ctsd, and Ctsb have been reported to be associated with pulmonary hypertension (3, 11). Ctsd, a marker of cellular senescence (37), is reportedly expressed in the in pulmonary vascular wall and is correlated with vascular remodeling (76). Given that their expression patterns correlated with phenotypic traits and elevated serum levels in irreversible PAH, these hub genes may be used as biomarkers reflecting PAH irreversibility.

## 5 Conclusion

In summary, the lysosome is dysfunctional in the development of irreversible PAH. The loss of compensation in lysosomal enzyme trafficking and sorting appears to be associated with a transition from reversible to irreversible PAH. Therefore, these results may provide new insights into the molecular mechanisms underlying irreversible PAH.

## Data Availability

The original contributions presented in the study are included in the article/Supplementary Material, further inquiries can be directed to the corresponding author/s.
