# Supplementary material for: Defect in lysosomal enzyme trafficking and sorting is associated with irreversibility of pulmonary arterial hypertension
Source: Front Cardiovasc Med. 2026 May 21;13:1763556. doi: 10.3389/fcvm.2026.1763556 (PMC13233543; doi:10.3389/fcvm.2026.1763556)
Supplement: Supplementary file 1 [file Datasheet1.docx]

Supplementary Material

## Supplementary Figures


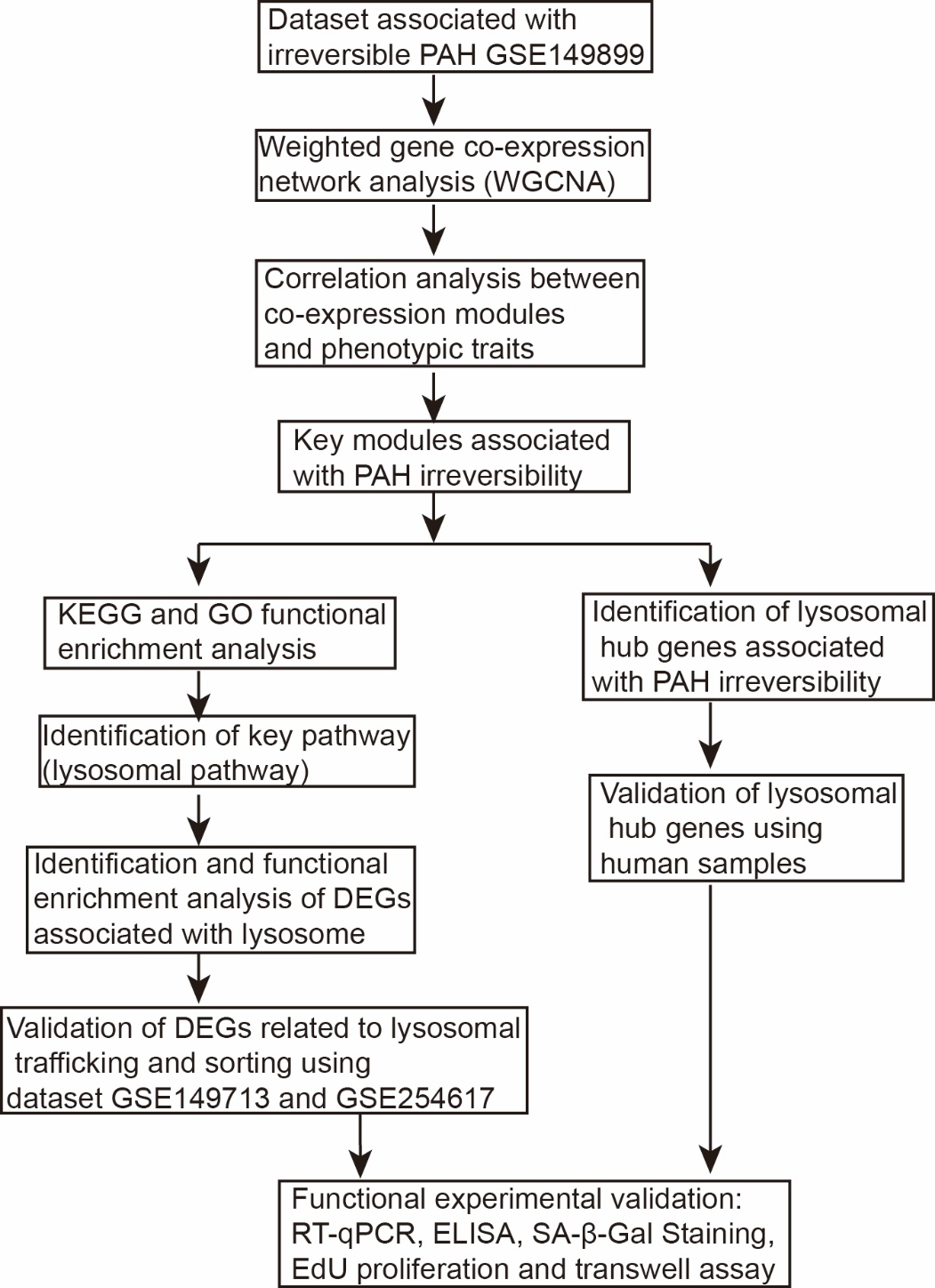


**Supplementary Figure 1.** The overall study workflow for this study


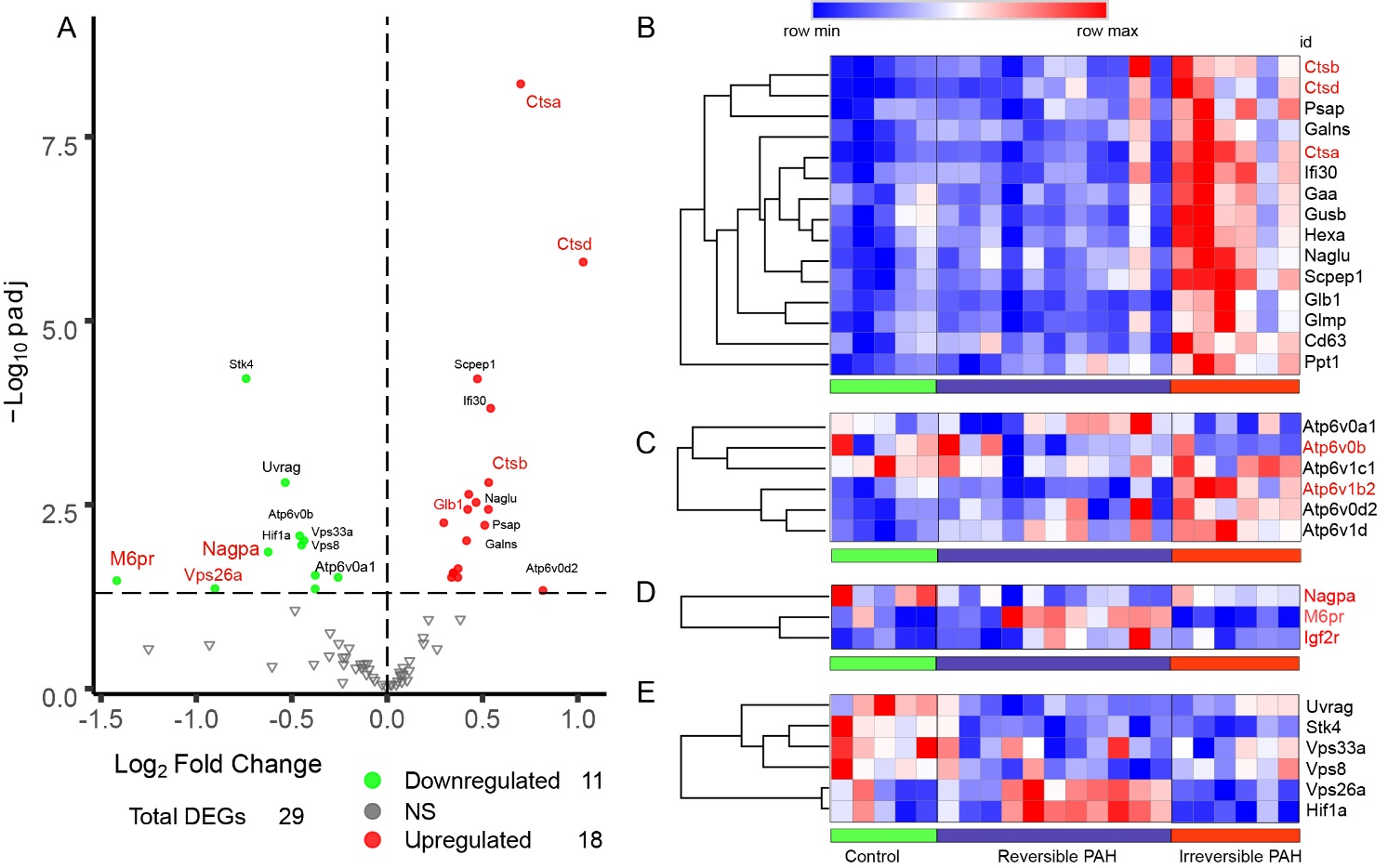


**Supplementary Figure 2.** Differential expression analysis of 73 genes with a well-known roles in lysosomal function. (A) Volcano plot showing the DEGs associated with lysosomal function in irreversible PAH. (B-E) Heatmaps illustrating DEGs related to lysosomal hydrolases and membrane proteins (B), lysosomal acidification (C), lysosomal biogenesis (D), and autophagy (E). TFEB target genes with known roles in lysosomal function were obtained from the study by Palmieri M et al. Hum Mol Genet 2011, 20:3852-3866.


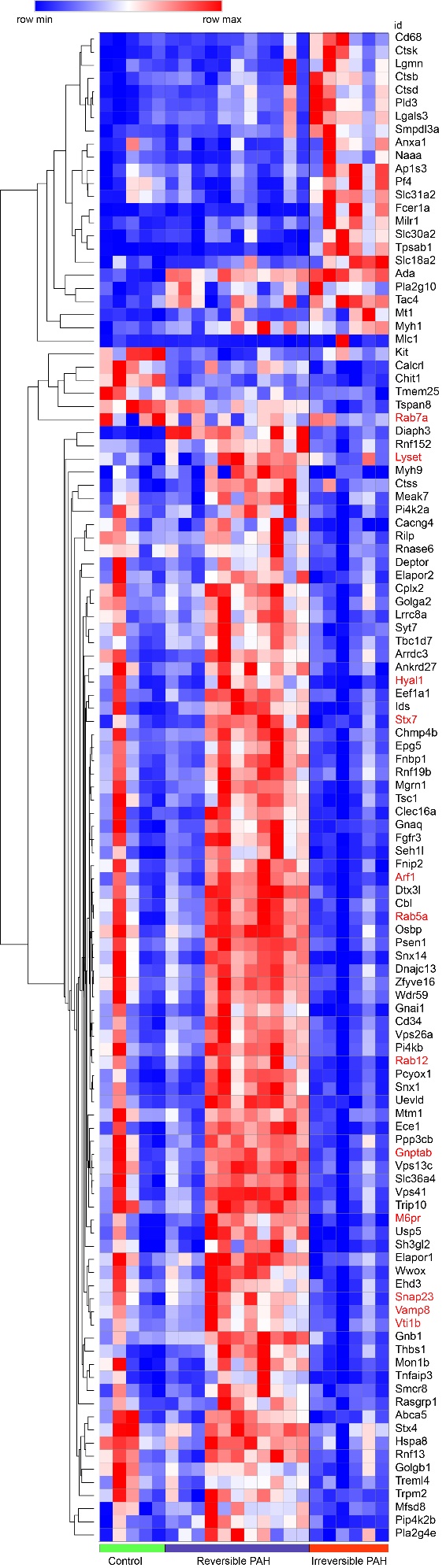


**Supplementary Figure 3.** Heatmap of all DEGs associated with lysosome. The rows in the heatmap represent gene expression levels, and columns represent each sample. DEGs were identified by using a threshold of log2Fold Change ≥ 1 and p-adj ≤0.05.


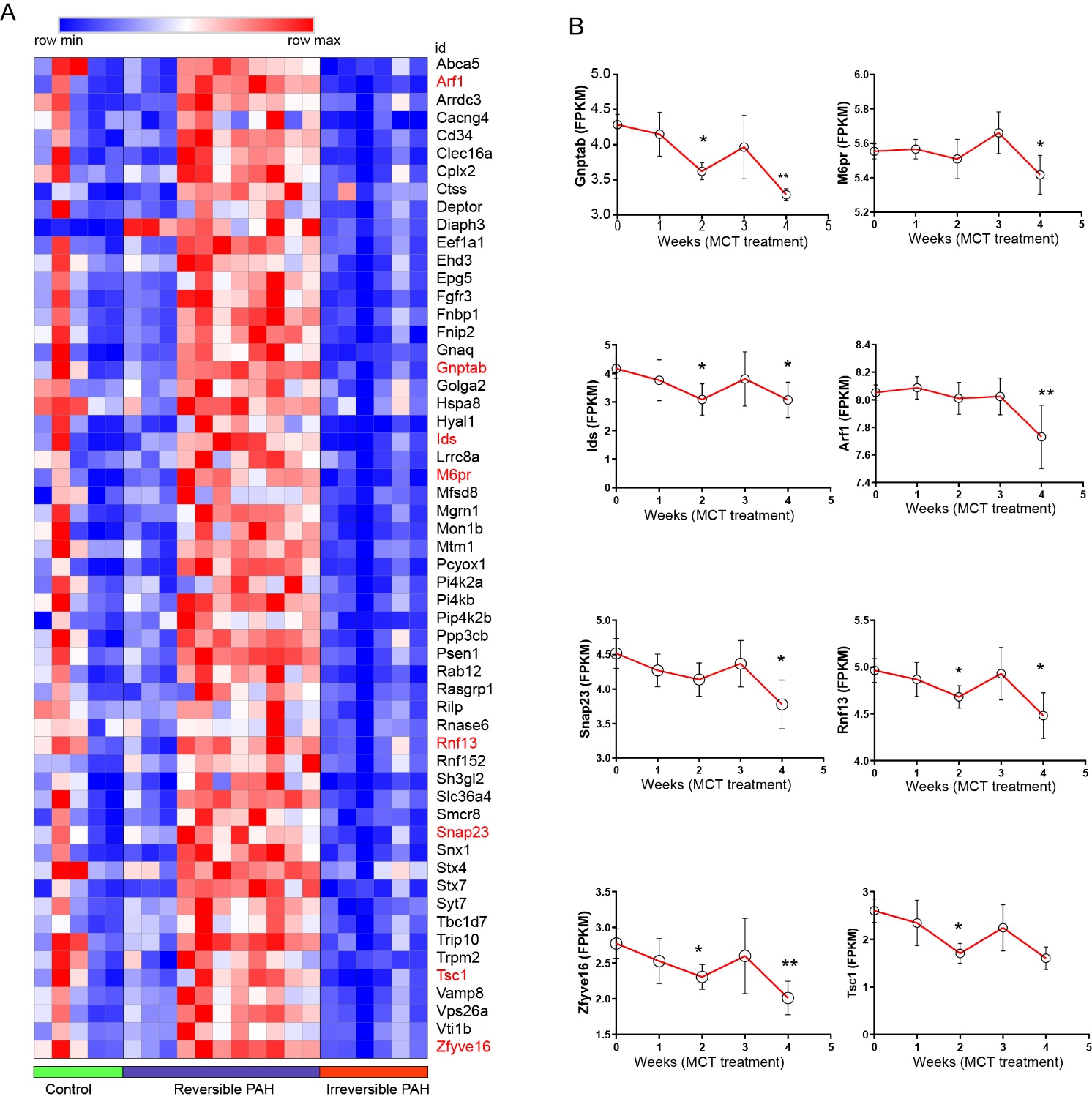


**Supplementary Figure 4.** DEGs involved in lysosomal trafficking and sorting. (A) Heatmap showing DEGs involved in lysosomal trafficking and sorting in MCT+shunt- induced PAH. (B) Validation of DEGs related to lysosomal enzyme trafficking and sorting in MCT-induced PAH. *p<0.05 vs control and **p<0.01 vs control. The DEGs were identified using a threshold of log2Fold Change ≥ 1 and p ≤0.05.


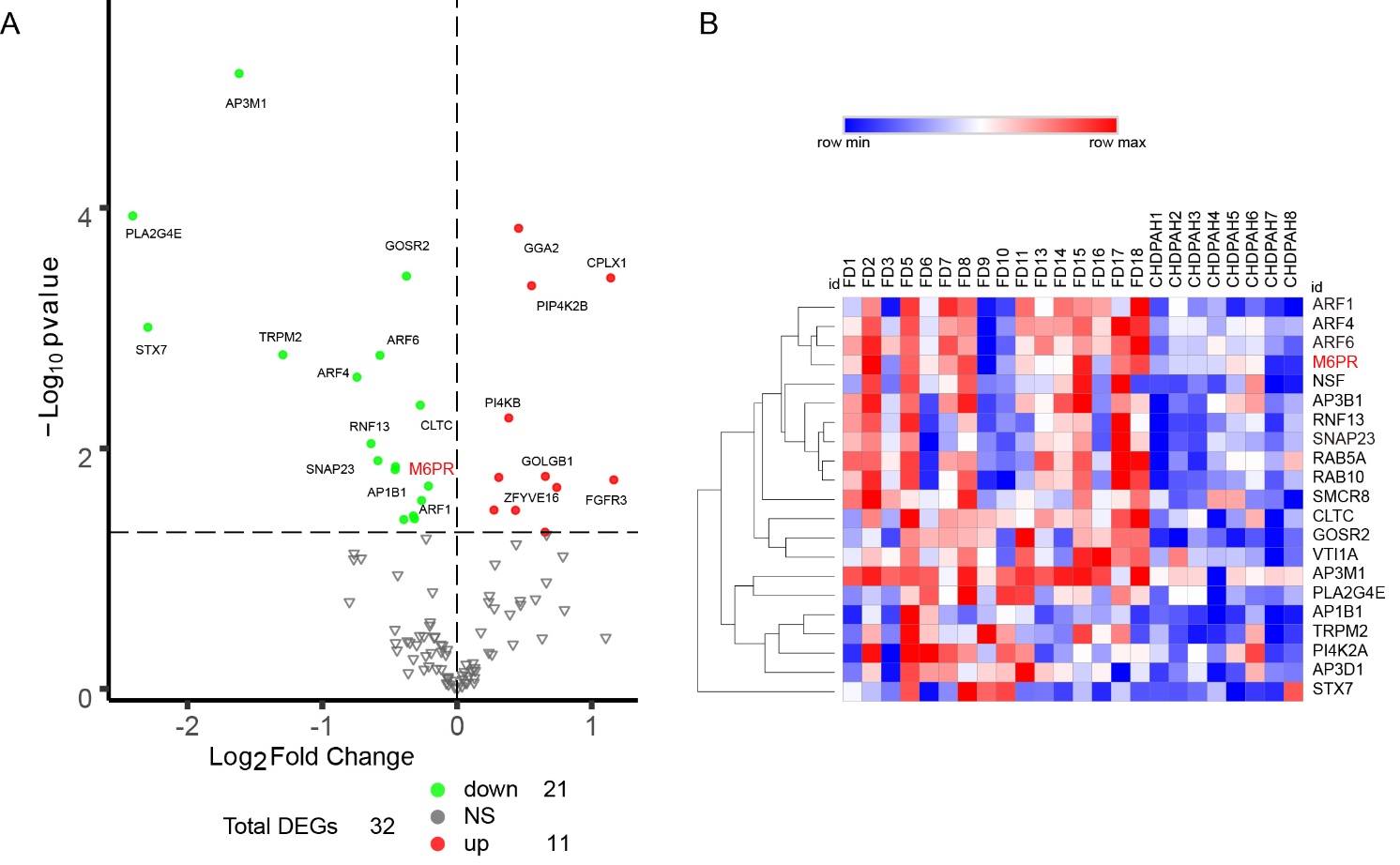


**Supplementary Figure 5.** Differential expression analysis of genes involved in lysosomal trafficking and sorting in congenital heart disease (CHD) patients with irreversible PAH. (A) Volcano plot displaying the DEGs involved in lysosomal trafficking and sorting in CHD patients with irreversible PAH. (B) Heatmap of downregulated genes associated with lysosomal trafficking and sorting in CHD patients with irreversible PAH. FD, failure donor (Control); CHD-PAH, congenital heart disease-associated pulmonary arterial hypertension.


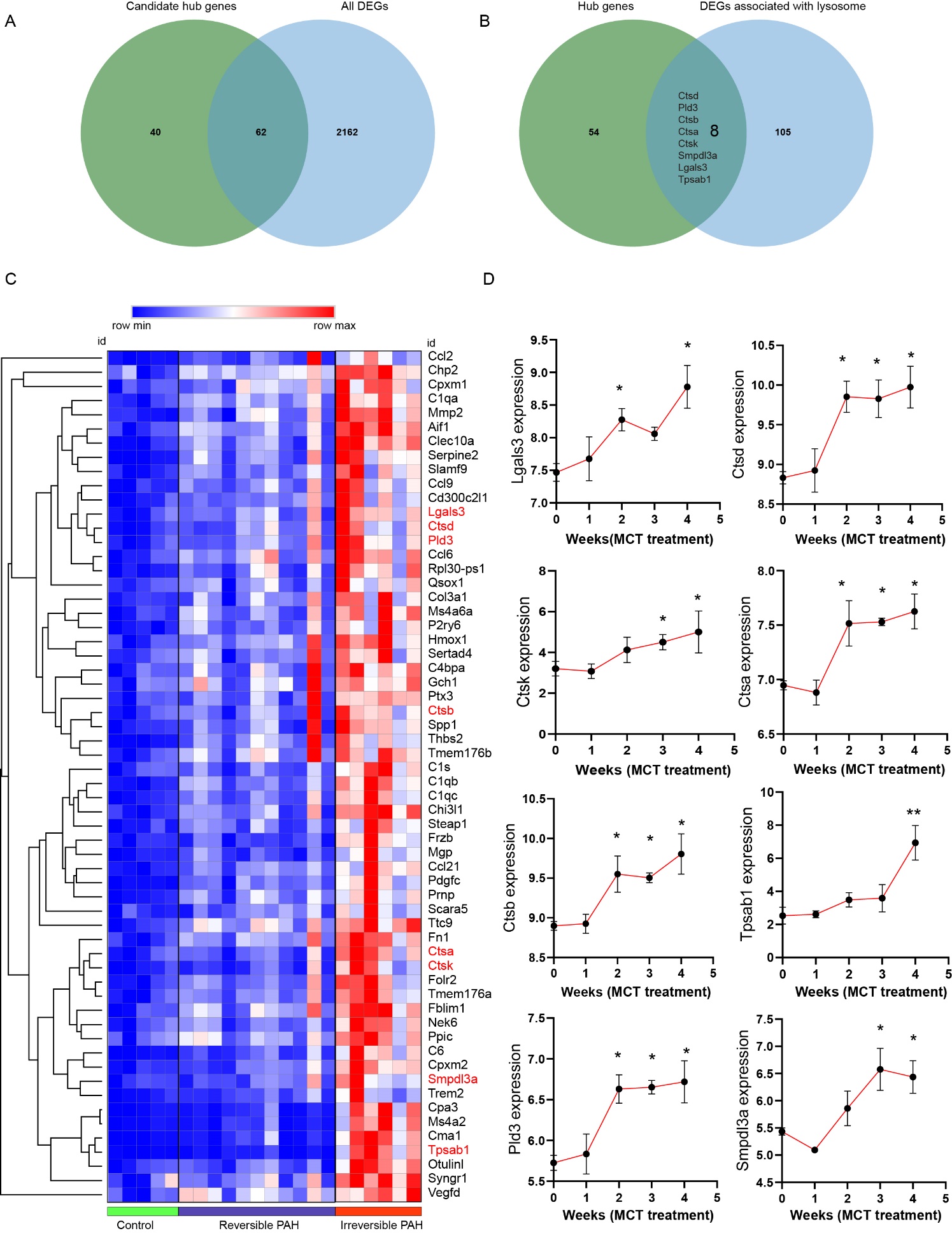


**Supplementary Figure 6.** **Identification of lysosomal hub genes associated with PAH irreversibility.** (A) Jvenn diagram showing 62 hub genes that were overlapped in candidate hub genes and all DEGs in MCT+shunt- induced PAH. (B) Jvenn diagram showing 8 lysosomal hub genes that were overlapped in hub genes and DEGs associated with lysosome. The overlapped genes were determined by using Jvenn software. (C) Heatmap showing 62 hub genes in MCT+shunt -induced PAH. (D) Temporal expression patterns of lysosomal hub genes in MCT-induced PAH. *p<0.05 vs control and **p<0.01 vs control. The DEGs were identified using a threshold of log2Fold Change ≥ 1 and p ≤0.05.

**Supplementary Table 2** Baseline characteristics of the study subjects

| Variables | CHD patients with reversible PAH  (n=20) | CHD patients with irreversible PAH  (n=20) | p value |
| --- | --- | --- | --- |
| Age | 48.25±15.22 | 47.65±18.21 | 0.9106 |
| Gender (female%) | 51.00 | 52.00 | 0.8506 |
| mPAP (mmHg) | 26.00±4.99 | 46.69±14.60 | 0.0002 |
| RVSP (mmHg) | 45.25±11.19 | 82.58±25.07 | 0.0003 |
| mRAP (mmHg) | 7.19±4.25 | 6.58±5.21 | 0.7461 |
| PVR (WU) | 3.28±1.25 | 6.01±2.34 | 0.0216 |
| Creatinine (mg/L) | 74.74±18.54 | 88.25±35.28 | 0.1424 |
| Uric acid (umol/L) | 341.53±89.48 | 464.00±182.05 | 0.0120 |
| eGFR(ml/min/1.73 m2) | 116.33±28.29 | 115.32±69.46 | 0.9535 |
| BUN(U/L) | 8.69±13.98 | 8.17±5.26 | 0.8811 |
| Sodium (mmol/L) | 140.71±1.93 | 138.47±4.27 | 0.0423 |
| Chloride (mmol/L) | 104.80±2.77 | 103.54±6.09 | 0.4088 |
| HGB(g/L) | 139.00±16.04 | 143.27±24.27 | 0.4324 |
| RDW-CV (%) | 13.15±1.23 | 13.92±2.2 | 0.2994 |
| PLT (10^9/L) | 233.90±43.50 | 179.89±76.38 | 0.0035 |
| PDW (%) | 13.13±3.14 | 13.92±2.8 | 0.4417 |
| PCT (%) | 0.23±0.06 | 0.19±0.07 | 0.0420 |
| MPV (fL) | 9.59±0.97 | 10.12±1.18 | 0.1747 |
| P-LCR (%) | 22.51±6.67 | 26.3±8.45 | 0.1957 |
